# Brainstem tuberculoma mimicking glioma in a high TB-endemic region: A diagnostic challenge and epidemiological alert: a case report

**DOI:** 10.1097/MD.0000000000050640

**Published:** 2026-09-11

**Authors:** Yuebu Amu, Laluo Sha Ma, Fei Xiao, Si Gao Deng, Yong Shi

**Affiliations:** a Department of Neurosurgery, The Second People’s Hospital of Liangshan Yi Autonomous Prefecture, Xichang, Sichuan, China.

**Keywords:** antituberculosis therapy, brainstem tuberculoma, case report, retrosigmoid approach

## Abstract

**Rationale:**

Brainstem tuberculomas account for < 0.2% of all intracranial tuberculomas and predominantly occur in children and adolescents; adult cases are extremely rare.

**Patient concerns:**

A 55-year-old male of Yi ethnicity presented with a 4-week history of progressive dizziness, gait instability, and left facial numbness. Brain magnetic resonance imaging revealed a 2.5 × 2.3 cm brainstem lesion with irregular rim enhancement and surrounding T2‑weighted fluid‑attenuated inversion‑recovery hyperintensity.

**Diagnoses:**

Brainstem space-occupying lesion, high suspicion of tuberculoma.

**Interventions:**

After 4 weeks of diagnostic antituberculosis therapy failed to achieve clinical improvement, gross total resection of the lesion was performed via a retrosigmoid approach, with intraoperative streptomycin irrigation.

**Outcomes:**

Histopathological examination confirmed tuberculous granuloma. The patient recovered well and received a standardized 12-month Isoniazid, Rifampicin, Pyrazinamide, and Ethambutol antituberculosis regimen postoperatively.

**Lessons:**

For patients with brainstem space-occupying lesions in tuberculosis high-burden regions, etiological examinations should be initiated early, even in the absence of typical systemic symptoms and positive serological findings. Surgical resection serves as an effective strategy to relieve mass effect for patients with severe space occupation.

## 1. Introduction

According to the World Health Organization Global Tuberculosis (TB) Report (2018), approximately 10.0 million individuals developed TB in 2017, with roughly 55% of cases concentrated in India, Indonesia, China, the Philippines, and Pakistan.^[1]^ Intracranial TB represents an uncommon complication of hematogenously disseminated TB, and brainstem involvement accounts for only 0.1 to 0.2% of all intracranial tuberculous lesions.^[2,3]^ Alongside improved living standards and widespread application of antiTB agents, its incidence has declined markedly, with 60 to 70% of cases occurring in children and adolescents. Fewer than 50 adult cases of brainstem tuberculoma have been documented worldwide.^[4]^ Herein, we report a case of a 55-year-old patient with brainstem tuberculoma to discuss diagnostic pitfalls and indications for surgical intervention.

## 2. Clinical data

### 2.1. Case history

A 55-year-old male of Yi ethnicity residing in a remote mountainous area was admitted with recurrent headache and dizziness lasting more than 1 month. He had resided locally for many years and denied fever, night sweats, cough, or expectoration.

### 2.2. Physical and auxiliary examinations

The patient was conscious but emaciated. Bilateral pupils were equal and round (approximately 3 mm in diameter) with brisk light reflexes. Left facial numbness was detected, accompanied by diminished superficial and deep sensation of the right hemibody. The right finger-to-nose test and Romberg sign were positive.

### 2.3. Laboratory and imaging findings

**Figure 1 and 2.**
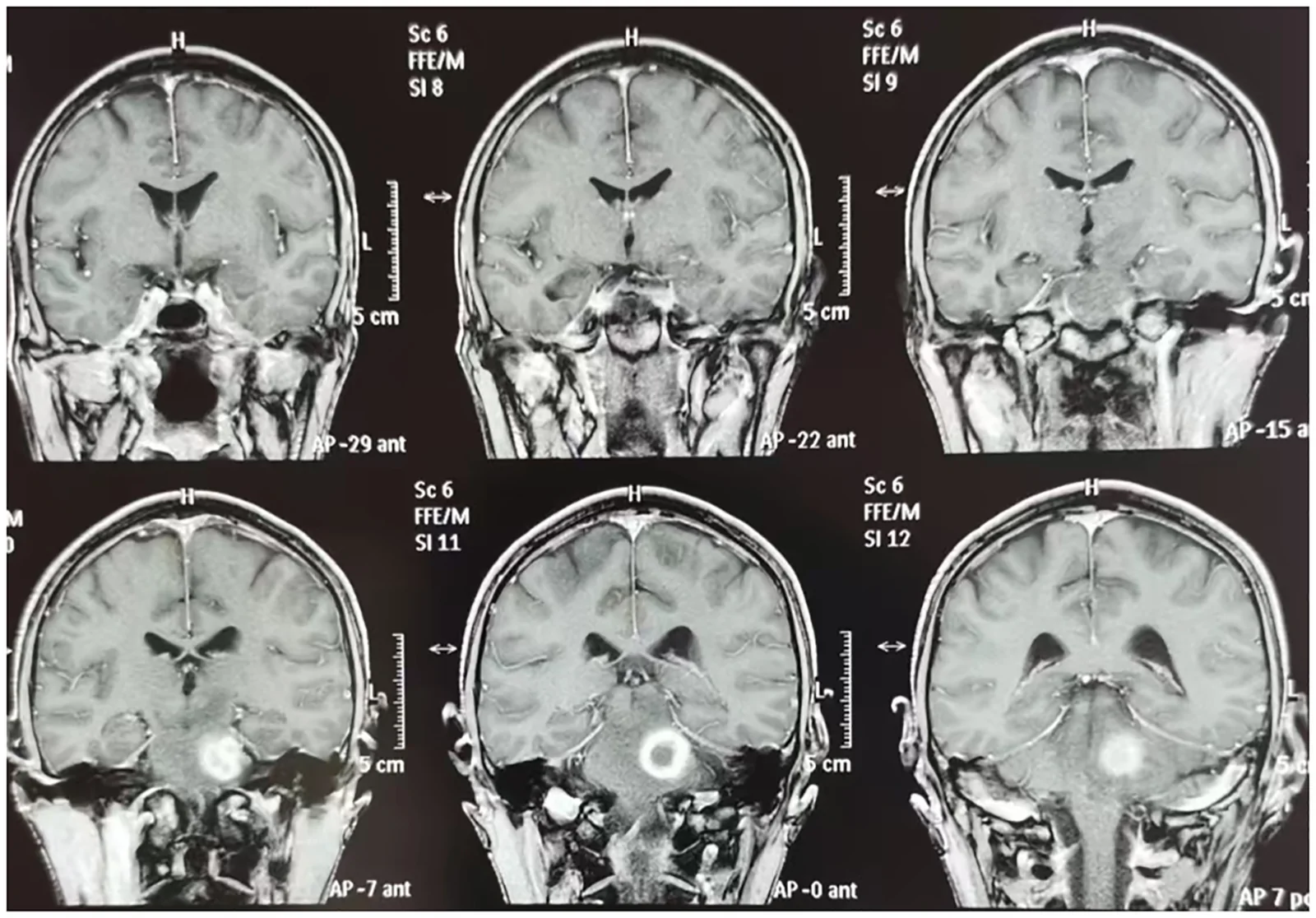

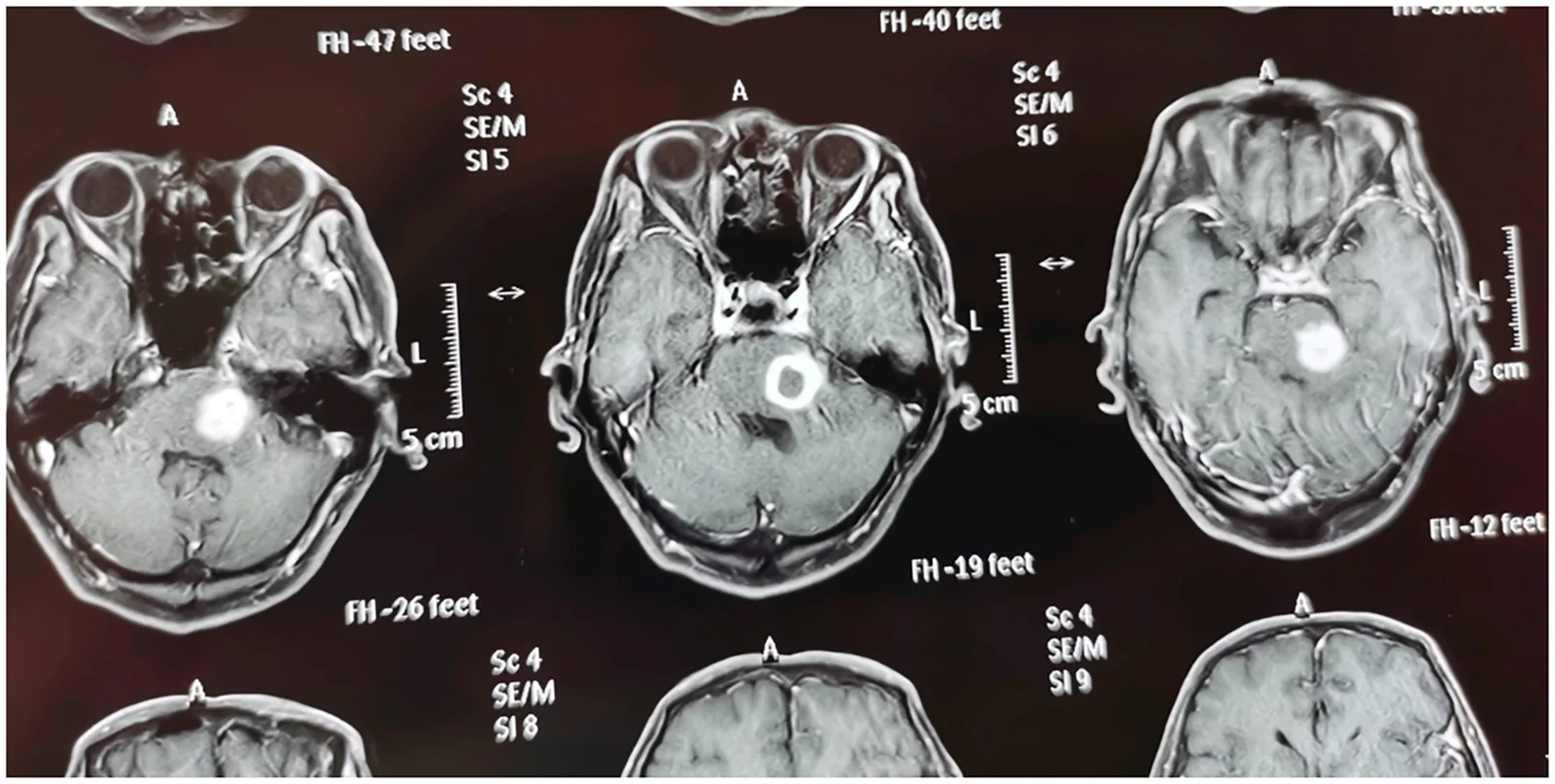
Brain MRI (plain + contrast-enhanced). Location: left pons. Signal characteristics: T1-weighted: isointense (isointense to brain parenchyma); T2-weighted: hypointense; DWI/FLAIR: mildly hypointense. Perilesional edema: prominent. Enhancement pattern: irregular enhancement without peripheral enhancement. MRI = magnetic resonance imaging.

Peripheral blood tests: white blood cell 6.76 × 10^9^/L, neutrophil percentage 63.4%, monocycle 8.3%. Cerebrospinal fluid analysis: adenine dreaminess 0.9 U/L, protein 1058 mg/L, glucose 2.93 mmol/L, chloride 125 mmol/L. Brain contrast-enhanced magnetic resonance imaging (MRI): An iso-T1, hypointense T2 lesion measuring 2.5 × 2.3 cm was identified within the left pons, with slight hypointensity on diffusion‑weighted imaging and fluid‑attenuated inversion‑recovery sequences. Severe perilesional edema was observed. The lesion displayed irregular enhancement without complete peripheral rim enhancement, highly suggestive of tuberculoma (Figs. 1 and 2). Chest computed tomography: scattered patchy, nodular and fibrous high-density shadows were visible in both lungs, consistent with suspected secondary pulmonary TB. Additional examinations: tuberculin purified protein derivative test: negative; sputum smear: no acid-fast bacilli; serum TB antibody: negative; cerebrovascular fluid TB antibody: negative; acid-fastbacilli culture: negative; *Mycobacterium tuberculosis* PCR: positive.

### 2.4. Diagnostic impressions

Brainstem space-occupying lesion: tuberculoma is highly suspected, while glioma cannot be excluded.

Suspected secondary pulmonary TB, pending further verification.

### 2.5. Treatment and clinical course

The patient was initiated on standard-dose antituberculosis (anti-TB) therapy:

- Isoniazid 0.3 g daily
- Rifampicin 0.45 g daily
- Ethambutol 0.75 g daily
- Pyrazinamide 1.5g daily

Supportive care included intensified dehydration management. After 4 weeks of anti-TB treatment, no significant clinical improvement was observed. Repeat cranial computed tomography demonstrated persistent mass effect without lesion regression.

During anti-TB treatment, the patient developed vomiting and persistent irritability, leading to poor treatment compliance. The patient strongly requested surgical resection and refused prolonged MRI protocols. Therefore, preoperative magnetic resonance spectroscopy (MRS) was not performed, and emergency lesion resection was carried out to alleviate mass effect on the brainstem.

## 3. Surgical indications and procedure

### 3.1. Surgical indications

The lesion caused prominent mass effect; clinical symptoms and imaging manifestations failed to improve after 4 weeks of anti-TB therapy. No surgical contraindications were identified, and both the patient and family actively requested surgical intervention.

### 3.2. Operative procedure

**Figure 3–5.**
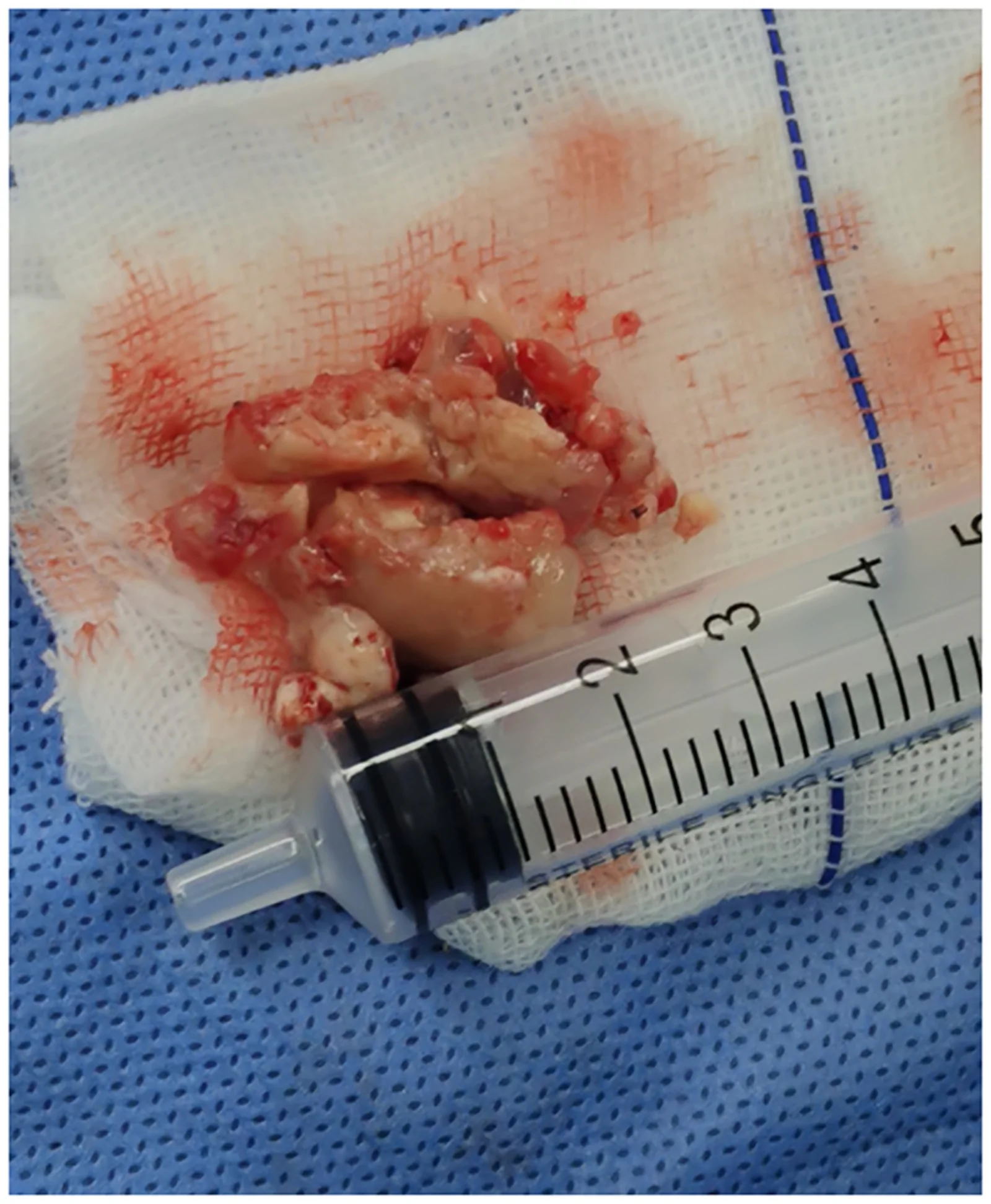

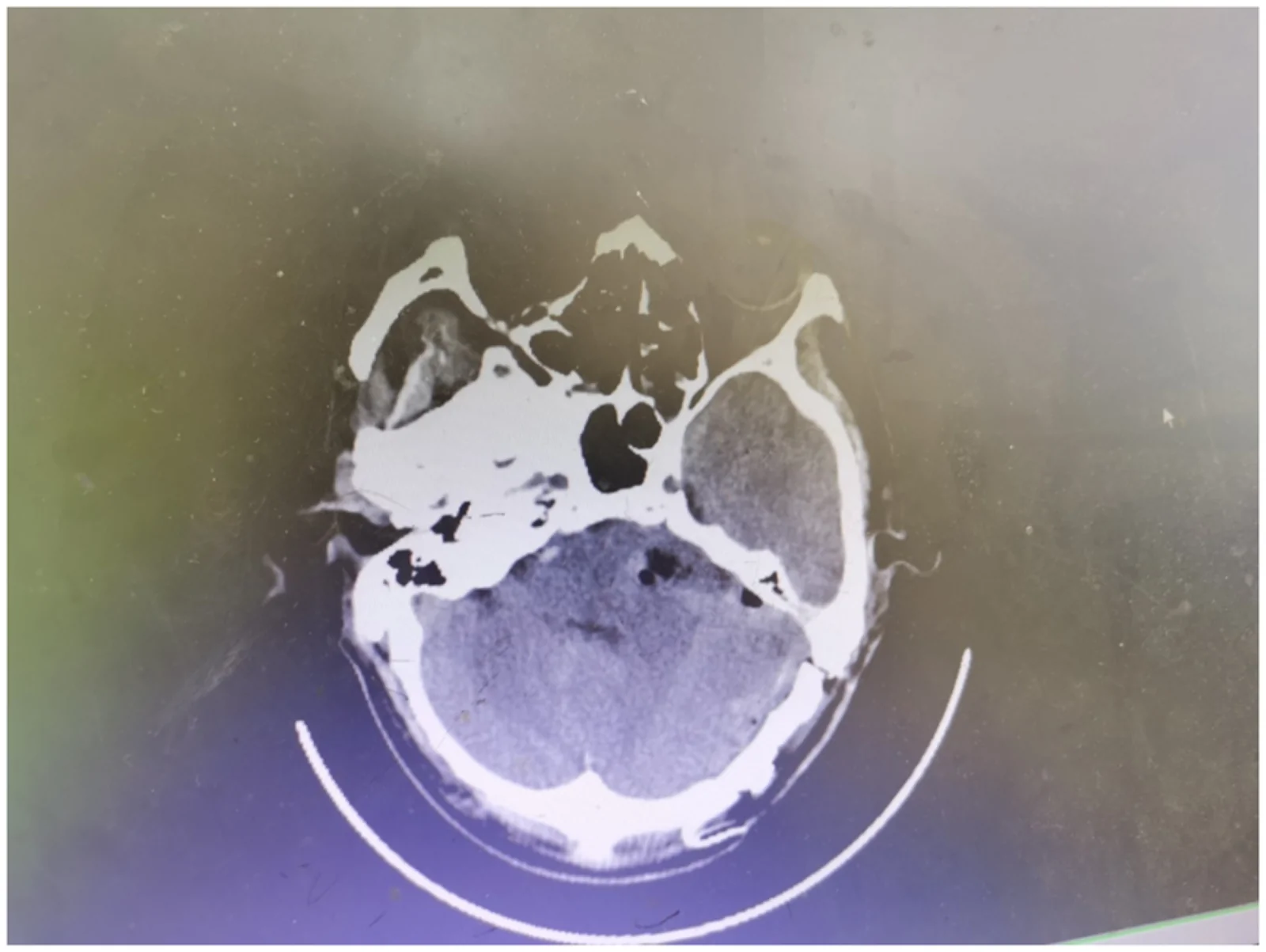

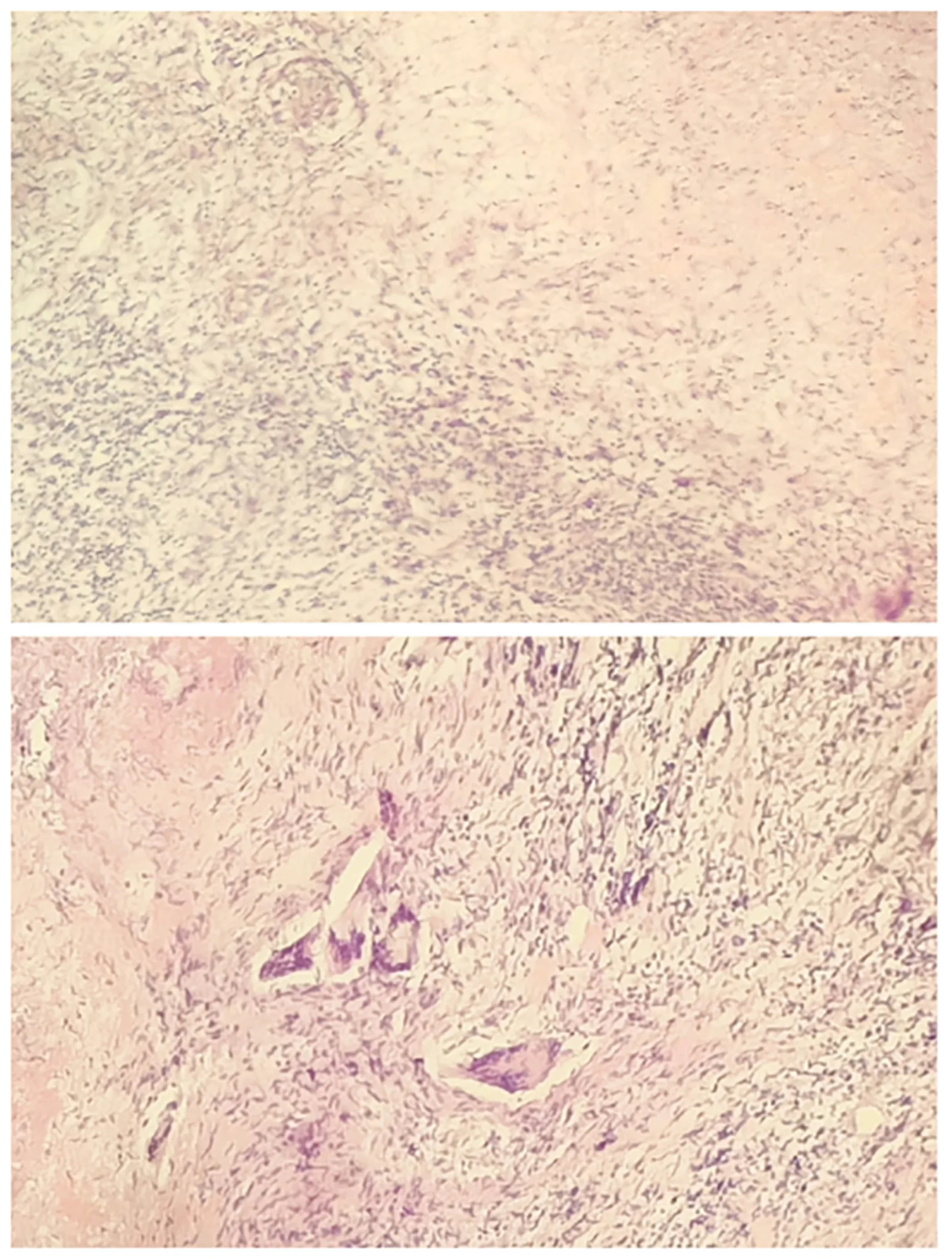
Surgical resection. The lesion was completely excised (gross total resection). Size: approximately 2.5 × 2.3 cm. Macroscopic appearance: necrotic tissue-like morphology. Postoperative evaluation: imaging follow-up showed satisfactory resection margins with no residual lesion. Pathological diagnosis: confirmed as tuberculous granuloma (consistent with preoperative radiological suspicion of tuberculoma).

A retrosigmoid approach was adopted for resection of the brainstem lesion. The lesion achieved gross total resection intraoperatively (Fig. 3).

### 3.3. Postoperative course

Postoperative imaging confirmed satisfactory resection without residual lesion (Fig. 4). The patient recovered uneventfully and was discharged. Histopathological examination verified brainstem tuberculous granuloma (Fig. 5).

Before surgery, the patient mainly complained of dizziness, fatigue, and limb numbness, without other associated neurological manifestations. After surgical resection, dizziness and fatigue were markedly relieved. Limb numbness partially improved during outpatient follow-up, and no new neurological deficits occurred. Standard anti-TB therapy was continued after discharge. Our institution does not require ethical approval for reporting individual cases or case series. Written informed consent was obtained from 1 or more legally authorized representatives for the publication of anonymized patient information in this article. All patient-identifiable details have been removed to preserve privacy.

## 4. Discussion

Intracranial tuberculoma, also termed intracranial tuberculous granuloma, refers to focal tuberculous infection within the brain parenchyma or meninges. Most lesions originate from hematogenous dissemination of tuberculous foci from other organs, forming microscopic granulomatous lesions (Rich foci) that subsequently aggregate into mature granulomas.^[2]^ A small subset arises as sequelae of diffuse tuberculous meningitis.^[3]^ Once hematogenously disseminated mycobacteria reach the central nervous system, macrophages, microglia, and astrocytes secrete multiple mediators that increase endothelial permeability, enabling bacteria to cross the blood-brain barrier.^[4]^ Tuberculoma and tuberculous meningitis are 2 devastating complications of systemic TB.^[4–7]^ In recent decades, improved living standards and widespread use of anti-TB medications have greatly reduced the incidence of cerebral TB. Nevertheless, 60 to 70% of cases still occur in children and adolescents, with a peak incidence before 5 years of age. Cases in infants younger than 3 months are rare, and the incidence is comparable between males and females.^[7]^ Our patient was a 55-year-old middle-aged male, representing an uncommon presentation of brainstem tuberculoma. Some clinicians recommend an initial trial of 4 to 8 weeks of anti-TB treatment; surgical intervention is reserved for patients without clinical improvement. Stereotactic biopsy remains the gold standard for definitive pathological diagnosis. Other scholars advocate anti-TB regimens ranging from 6 to 36 months, while primary surgical resection may be considered for lesions larger than 20 mm.^[8]^

In our case, the patient suffered severe symptoms and exhibited no subjective improvement following 4 weeks of anti-TB therapy, and strongly favored surgical resection. Gross total resection was therefore performed. The lesion was carefully dissected and removed en bloc in 2 fragments. No cystic fluid or purulent secretion was identified within the lesion cavity. Intraoperative irrigation with streptomycin (0.5 mg/mL) was administered to reduce the risk of mycobacterial dissemination. The patient achieved favorable postoperative recovery and was discharged, reaching the expected therapeutic outcome. Brainstem tuberculoma remains rare and creates major diagnostic challenges.^[9]^ For high-risk populations including ethnic minorities, migrants, malnourished individuals, and immunocompromised patients presenting with unexplained brainstem lesions, clinicians should maintain a high suspicion of tuberculoma even when initial TB laboratory tests return negative. In regions with high TB prevalence, tuberculoma should be prioritized in the differential diagnosis over glioma.^[10]^ Our patient was an ethnic Yi resident from remote mountainous Liangshan, an area with high endemic TB transmission facilitated by local living customs such as shared tableware, which further supported the clinical suspicion of tuberculoma.

MRI serves as a fundamental imaging modality for suspected cerebral tuberculoma in TB-endemic areas. MRS provides supplementary information for differential diagnosis between tuberculoma and other intracranial masses. Typical MRS features of tuberculoma include minimal amino acid peaks and elevated lactate (distinguishing it from pyogenic abscess), alongside a choline/creatinine ratio > 1 (differentiating from neurocysticercosis).^[11]^

Preoperative MRS was not acquired in this case. After 4 weeks of anti-TB therapy, the patient failed to achieve clinical improvement and developed drug-induced vomiting and persistent irritability. The patient demonstrated poor compliance and insisted on surgical resection while refusing prolonged MRI examinations. Given progressive neurological deterioration, emergency resection was prioritized to relieve brainstem mass effect rather than completing MRS. We acknowledge the absence of preoperative MRS as one limitation of this case report.

Stereotactic biopsy enables definitive diagnosis and facilitates timely initiation of anti-TB therapy, yielding favorable outcomes in most patients. Patients with tuberculoma require regular contrast-enhanced MRI surveillance throughout treatment. However, consensus regarding optimal treatment duration remains lacking. The American Thoracic Society and Centers for Disease Control and Prevention recommend a 12-month regimen for drug-susceptible intracranial TB. For symptomatic patients with prominent mass effect, combined gross total surgical resection and anti-TB therapy constitutes the optimal management strategy.

## 5. Conclusions

Warning for high-risk populations: for brainstem space-occupying lesions in TB-endemic regions such as Liangshan, China, tuberculoma should be ranked high in the differential diagnosis even without typical systemic tuberculous manifestations.

Clinical value of surgery: early lesion resection via the safe and effective retrosigmoid approach can reverse progressive neurological deficits in patients with significant mass effect.

Standardized diagnostic and therapeutic workflow: establish a systematic management pathway combining etiological testing, multimodal imaging assessment, and individualized surgical decision-making.

## Acknowledgements

We sincerely thank all radiology and pathology staff for their professional technical support, which laid a solid foundation for this case report. We also appreciate assistance provided by other participating colleagues and institutions.

## Author contributions

**Conceptualization:** Laluo Sha Ma.

**Data curation:** Yuebu Amu, Laluo Sha Ma.

**Resources:** Yuebu Amu, Fei Xiao.

**Software:** Yuebu Amu.

**Supervision:** Fei Xiao, Si Gao Deng.

**Validation:** Yong Shi.

**Visualization:** Yong Shi.

**Writing** – **original draft:** Yuebu Amu.

**Writing** – **review & editing:** Yuebu Amu.
